# Bigram-PGK: phosphoglycerylation prediction using the technique of bigram probabilities of position specific scoring matrix

**DOI:** 10.1186/s12860-019-0240-1

**Published:** 2019-12-20

**Authors:** Abel Chandra, Alok Sharma, Abdollah Dehzangi, Daichi Shigemizu, Tatsuhiko Tsunoda

**Affiliations:** 1 School of Engineering and Physics, Faculty of Science Technology and Environment, University of the South Pacific, Suva, Fiji; 2 Institute for Integrated and Intelligent Systems, Griffith University, Brisbane, QLD 4111 Australia; 3 Department of Medical Science Mathematics, Medical Research Institute, Tokyo Medical and Dental University (TMDU), Tokyo, 113-8510 Japan; 4 Laboratory for Medical Science Mathematics, RIKEN Center for Integrative Medical Sciences, Yokohama, Kanagawa 230-0045 Japan; 5 CREST, JST, Tokyo, 102-8666 Japan; 6 Department of Computer Science, Morgan State University, Baltimore, MD USA; 7 Medical Genome Center, National Center for Geriatrics and Gerontology, Obu, Aichi 474-8511 Japan; 8 Laboratory for Medical Science Mathematics, Department of Biological Sciences, Graduate School of Science, The University of Tokyo, Tokyo, 108-8639 Japan

**Keywords:** Post-translational modification, Phosphoglycerylation, Lysine residue, Computational technique, Evolutionary information

## Abstract

**Background:**

The biological process known as post-translational modification (PTM) is a condition whereby proteomes are modified that affects normal cell biology, and hence the pathogenesis. A number of PTMs have been discovered in the recent years and lysine phosphoglycerylation is one of the fairly recent developments. Even with a large number of proteins being sequenced in the post-genomic era, the identification of phosphoglycerylation remains a big challenge due to factors such as cost, time consumption and inefficiency involved in the experimental efforts. To overcome this issue, computational techniques have emerged to accurately identify phosphoglycerylated lysine residues. However, the computational techniques proposed so far hold limitations to correctly predict this covalent modification.

**Results:**

We propose a new predictor in this paper called Bigram-PGK which uses evolutionary information of amino acids to try and predict phosphoglycerylated sites. The benchmark dataset which contains experimentally labelled sites is employed for this purpose and profile bigram occurrences is calculated from position specific scoring matrices of amino acids in the protein sequences. The statistical measures of this work, such as sensitivity, specificity, precision, accuracy, Mathews correlation coefficient and area under ROC curve have been reported to be 0.9642, 0.8973, 0.8253, 0.9193, 0.8330, 0.9306, respectively.

**Conclusions:**

The proposed predictor, based on the feature of evolutionary information and support vector machine classifier, has shown great potential to effectively predict phosphoglycerylated and non-phosphoglycerylated lysine residues when compared against the existing predictors. The data and software of this work can be acquired from https://github.com/abelavit/Bigram-PGK.

## Background

The biological process of enzymatic change in proteins brought about after the translation in the ribosome is known as post-translational modification (PTM). The high interest in PTM for various organisms have emerged as a result of efforts in high-throughput proteomics for the study of site-specific PTM as well as enzymes which cause these modifications [1]. The genetic code comprises 20 amino acids and out of which, lysine is the most commonly modified [2, 3]. From the literature [4], some of the major covalent modifications of lysine residues are acetyl [5], glycosyl [6], methyl [7], succinyl [8], pupyl [9], crotonyl [10], and propionyl [11]. These various amino acid modifications, as well as their regulatory enzymes, are associated with several human diseases including heart disease, rheumatoid arthritis, multiple sclerosis, neurodegenerative disorders, and celiac disease [12–15].

Phosphoglycerylation, which is a non-enzymatic lysine modification, is a type of PTM that has been recently discovered in human cells and mouse liver [16, 17]. Cardiovascular disease, such as heart failure, is a highly probable condition caused by phosphoglycerylation since this chemical modification is associated with glycolytic pathways and glucose metabolism [18, 19]. Phosphoglycerylation is dynamic and reversible and occurs as a result of reaction between primary glycolytic intermediate (1,3-BPG) and lysine residue, which result in the formation of 3-phosphoglyceryl-lysine (pgK) [17]. Glycolytic enzymes are affected by pgk. It also builds up on cells having high exposure to glucose. As a result, potential feedback mechanism which leads to build up and redirection of glycolytic intermediates to different biosynthetic pathways is established. As this PTM is relatively new to the field, it is important to identify and analyze its functional aspects to be able to understand the selectivity mechanism and its regulatory roles for better diagnosis and treatment of affected persons.

The method of computational techniques to identify phosphoglycerylation sites is getting popular nowadays [20–34] as the pure experimental methods, such as mass spectrometry, is quite inefficient, time-consuming and at the same time expensive [35–37]. The promising results of computational techniques has resulted in great confidence to identify phosphoglycerylated and non-phosphoglycerylated sites over the traditional method.

In the recent years, there have been a number of studies involved to identify phosphoglycerylation using computational technique. Phogly-PseAAC was the earliest work to be carried out where it utilized a KNN-based predictor to predict phosphoglycerylation using a feature set of pseudo amino acid composition [38]. The second work called CKSAAP_PhoglySite uses the composition of k-spaced amino acid pairs (CKSAAP) as features and employs a fuzzy support vector machine to predict [16]. Finally, the recent work named iPGK-PseAAC was proposed and it uses a four tier amino acid pairwise coupling technique alongside a SVM operation engine for prediction [39].

The proposed predictors of phosphoglycerylation in the literature are still limited in terms of their performance. In this regard, we are introducing a novel predictor called Bigram-PGK which employs evolutionary information to predict phosphoglycerylated and non-phosphoglycerylated lysine residues. A total of 91 protein sequences were used in this work which contained experimentally confirmed phosphoglycerylated sites and their profile bigram was obtained from the position-specific scoring matrix (PSSM). With the evolutionary information of the sequences, different segment sizes for each lysine residue was analyzed in terms of the performance. The residue window of ±32 performed the best on Mathews correlation coefficient (MCC) metric when the size of ±1 to ±32 was considered (see Additional file 1). Residue window sizes further than ±32 could not be taken into account due to constrain of the protein sequence length. Hence a lysine residue, whether phosphoglycerylated or non-phosphoglycerylated, was considered by encompassing a stretch of 32 upstream and 32 downstream amino acids to the lysine with the lysine residue at the center. The number of phosphoglycerylated residues were small compared to the non-phosphoglycerylated, therefore a k-nearest neighbors cleaning treatment was implemented to deal with the class imbalance [35, 40, 41]. The balanced dataset was then used to construct the Bigram-PGK predictor using LibSVM package which showed a superior performance over the existing methods on the 10-fold cross-validation procedure. The performance of Bigram-PGK on the metrics sensitivity, specificity, precision, accuracy, MCC and area under the ROC curve (AUC) was 0.9642, 0.8973, 0.8253, 0.9193, 0.8330, 0.9306, respectively.

## Results and discussion

### Dataset balancing

The phosphoglycerylation dataset obtained from PLMD was found to be imbalanced, whereby the phosphoglycerylated sites were much less compared to that of non-phosphoglycerylated. The 111 phosphoglycerylated sites compared to 3249 non-phosphoglycerylated sites resulted in an imbalance ratio of 1:29. Having imbalance ratio of this magnitude will easily bias the classification process. Resolving the class imbalance is critical in order to build a reliable predictor. To deal with the imbalance issue, we employed the commonly used k-nearest neighbor cleaning treatment which removed instances from the majority class (non-phosphoglycerylated in this case) when they are one of the k neighbors of a positive instance (phosphoglycerylated site) [35, 37, 40, 42, 43]. The cleaning treatment was initiated with a k value equal to the imbalance ratio i.e. 29. The intention was to remove those negative instances which were among the 29 neighbors of every positive instance. With a k value of 29, the imbalance ratio remained undesirable hence the threshold was further increased until the final data set attained an imbalance ratio of 1:2. As a result, the number of non-phosphoglycerylated sites was reduced to 224 instances after applying a k value of 111. The final dataset of 111 positive instances and 224 negative instances, obtained using a k value of 111, was used to validate the performance of the predictor.

### Statistical measures

In proposing any new predictor, it is crucial to assess its performance. In this work, we have employed five statistical measures including sensitivity, specificity, precision, accuracy, and Mathews correlation coefficient [16, 35, 38, 41, 42, 44–47]. Furthermore, we have calculated the area under the ROC curve of the predictor and it is depicted in the later section.

The first metric, sensitivity, determines the ability of the classifier to correctly predict phosphoglycerylated lysine sites. The measure ranges from 0 to 1 where a higher value indicates the better the predictor is in classifying the phosphoglycerylated sites. Specificity is the second metric and it measures the ability of the classifier to correctly predict non-phosphoglycerylated lysine sites. This metric also takes on the 0 to 1 range of values where a high value indicates that the predictor is effective at predicting non-phosphoglycerylated sites. The third and fourth metrics are precision and accuracy, respectively, and they take on the same range of values as sensitivity and specificity. Precision metric assesses capability of the predictor to correctly classify phosphoglycerylated sites from all the phosphoglycerylated sites predicted. The accuracy metric evaluates how correctly the predictor distinguishes between phosphoglycerylated sites and non-phosphoglycerylated sites. Mathews correlation metric, which is the fifth measure, assesses the quality of the predictor. The range of values of MCC metric is − 1 to + 1 where − 1 signifies a completely negative correlation, while + 1 indicates a highly positive correlation. These five statistical measures can be written as equations as shown below:

1 $$ Sensitivity=\frac{TP}{TP+ FN} $$

2 $$ Specificity=\frac{TN}{TN+ FP} $$

3 $$ Precision=\frac{TP}{TP+ FP} $$

4 $$ Accuracy=\frac{TN+ TP}{FN+ FP+ TN+ TP} $$

5 $$ MCC=\frac{\left( TN\times TP\right)-\left( FN\times FP\right)}{\sqrt{\left( TP+ FP\right)\left( TP+ FN\right)\left( TN+ FP\right)\left( TN+ FN\right)\ }} $$

In the equations above, FN, FP, TN, and TP represents false negatives, false positives, true negatives and true positives, respectively. False negatives represents instances which are phosphoglycerylated sites but predicted as non-phosphoglycerylated. False positives are those that are non-phosphoglycerylated sites yet predicted as phosphoglycerylated. The true negatives are instances correctly predicted as non-phosphoglycerylated sites and finally, true positives are instances correctly predicted as phosphoglycerylated sites. It is desirable for the best predictor to have high scores in all of the statistical measures. Nevertheless, the proposed predictor should at least have higher sensitivity measure compared to the existing predictors.

### Validation scheme

The statistical measures outlined in the previous section to assess the predictor’s performance was carried out using the 10-fold cross-validation scheme. In the literature, there are three common ways of determining the effectiveness of a predictor and these are n-fold cross-validation test, independent dataset test, and the jackknife test [48, 49]. Though the jackknife test is regarded to be the least arbitrary of the three and outputs distinctive result on dataset [50], we employed the n-fold cross-validation scheme to avoid high computational time, with n equal to 10. The below steps highlight the 10-fold cross-validation procedure:

- Step 1: Divide the dataset into 10 equal parts
- Step 2: Train predictor by combining the 9 parts and test it using the part left out
- Step 3: Adjust the classifier parameters with training set
- Step 4: Obtain the statistical measures with the test set
- Step 5: Repeat steps 2 to 4 until all the folds have been used as test sets and average the statistical measures

The result of 10-fold cross-validation scheme on Bigram-PGK is presented in the following section.

### Bigram-PGK comparison with available predictors

There are three predictors in the literature which carry out the classification of phosphoglycerylated and non-phosphoglycerylated sites, and these are Phogly_PseAAC [38], CKSAAP_PhoglySite [16], and iPGK_PseAAC [39]. Firstly, we obtained the predictions of these methods on all the lysine residues in our benchmark dataset. This was carried out by preparing the dataset in FASTA format and uploading it to the webservers of Phogly-PseAAC and iPGK-PseAAC predictors, and for the CKSAAP_PhoglySite predictor by inputting the file to the Matlab software package. It is intuitive to point out that these predictors may have been trained using samples which are being used to carry out the performance evaluation and therefore the results can be biased in their favor. The performance comparison of our predictor against the existing methods was carried out on the validation set, the sets put aside as test sets during the 10-fold cross-validation scheme. Likewise, the same validation set was used to obtain the performance of the other methods by investigating their predictions on those samples when the benchmark dataset was uploaded to the respective webserver/software packages.

The comparison result of Phogly_PseAAC [38], CKSAAP_PhoglySite [16], iPGK_PseAAC [39], and our predictor Bigram-PGK is shown in Table 1. In Table 1, we have also added the AUC measure for all the predictors for a more robust comparison since the predictor with the highest AUC measure is always favorable. It can be seen from the results that Bigram-PGK gives the highest performance on the metrics sensitivity, accuracy, MCC and AUC. The sensitivity measure increased by 16.4%, accuracy by 1.7%, MCC by 6.5%, and AUC by 5.1%. These improved performances goes on to say that Bigram-PGK is quite an effective predictor for the phosphoglycerylation problem. From Table 1, it can also be realized that iPGK_PseAAC predictor [39] obtained the highest specificity measure (0.9864) but its sensitivity measure is very low (0.4555), which shows that almost 55% of the phosphoglycerylated sites were undetected by this method.

**Table 1:** Comparison of the three existing prediction methods with the Bigram-PGK predictor using 10-fold cross-validation scheme

Predictor	Sensitivity	Specificity	Precision	Accuracy	MCC	AUC
iPGK_PseAAC [39]	0.4555	**0.9864**	**0.9548**	0.8119	0.5692	0.7230
CKSAAP_PhoglySite [16]	0.8285	0.9420	0.8765	0.9043	0.7818	0.8854
Phogly_PseAAC [38]	0.6927	0.7193	0.5518	0.7102	0.3951	0.7062
Bigram-PGK	**0.9642**	0.8973	0.8253	**0.9193**	**0.8330**	**0.9306**

Highest values of the metrics are highlighted in bold

The promising result in Table 1 clearly illustrate the ability of Bigram-PGK to correctly predict phosphoglycerylated and non-phosphoglycerylated lysine residues. This can be credited to the use of underlying important evolutionary information in protein sequences. The information is captured in PSSM of amino acids surrounding the lysines and when this information is transformed into the matrix of bigram occurrences, it produces the necessary characteristics for identifying the modified lysines. Furthermore, the improved performance can also be attributed to the SVM algorithm for its effective data handling.

### Insights into phosphoglycerylation prediction

In the Additional file 2, we present the analysis of phosphoglycerylation sites predicted by iPGK_PseAAC [39], CKSAAP_PhoglySite [16], Phogly_PseAAC [38], and Bigram-PGK on the 10-fold cross-validation procedure. It has been observed that for the proteins having multiple phosphoglycerylation sites, not all the predictors were able to detect them entirely. In fact, only the Bigram-PGK predictor managed to detect almost all of these proteins. The only protein which went undetected was Beta-globin (UniProt Accession A8DUK4) which is a subunit of a larger protein named hemoglobin [51], and this protein was successfully identified by the Phogly_PseAAC [38] predictor alone. Moreover, Bigram-PGK was the only one that effectively detected all phosphoglycerylation sites of the protein Carbamoyl-phosphate synthase (UniProt Accession Q8C196) which plays a vital role in the removal of surplus ammonia from the cell of ureotelic animals [52]. Moving on to the proteins with single phosphoglycerylation site, there were a number of proteins which only the Bigram-PGK predictor was able to detect. These proteins include Arf-GAP with SH3 domain (UniProt Accession E9QMI7) which regulates the formation of post-Golgi vesicles and controls constitutive secretion [53], 14–3-3 protein beta/alpha (UniProt Accession A2A5N1) which regulates both general and specialized signaling pathways [54], 60S ribosomal protein L31 (UniProt Accession P62900) which is heavily involved in RNA binding and structural integrity of the ribosome [55], and Zinc finger protein GLI1 (UniProt Accession P47806) which acts as a transcriptional activator [56]. There were also proteins that the Bigram-PGK could not detect but were detected by the rest of the predictors. These proteins include Glutamate receptor ionotropic (UniProt Accession B1AS29) which acts as an excitatory neurotransmitter at many synapses in the central nervous system [57], and EH domain-containing protein 4 (UniProt Accession Q9EQP2) that binds ATP and membrane and it could likely control membrane reorganization upon ATP hydrolysis [58]. Furthermore, none of the predictors were able to detect phosphoglycerylation site of a couple of the proteins. One such protein is Proline synthase co-transcribed bacterial homolog protein (UniProt Accession Q80ZV3) which selectively and non-covalently interacts with biologically active form of vitamin B6 and other vitamin B elements [59]. Nevertheless, all the predictors successfully predicted the phosphoglycerylation site of many of the proteins such as ATP-dependent 6-phosphofructokinase (UniProt Accession P47857) which acts as a catalyst in phosphorylation of D-fructose 6-phophate to fructose 1,6-bisphosphate by ATP [60], Farnesyl pyrophosphate synthase (UniProt Accession Q920E5) which plays a key role in isoprenoid biosynthesis [61], Calcium-binding mitochondrial carrier protein Aralar2 (UniProt Accession Q9QXX4) which acts as a catalyst in calcium-dependent exchange of cytoplasmic glutamate with mitochondrial aspartate [62], Triosephosphate isomerase (UniProt Accession P60174) that catalyzes interconversion between dihydroxyacetone phosphate and D-glyceraldehyde-3-phosphate in glycolysis [63], Kinectin (UniProt Accession Q86UP2) which is involved in kinesin-driven vesicle motility [64], Fructose-bisphosphate aldolase (UniProt Accession A6ZI44) which plays an important role in glycolysis and gluconeogenesis [65], and Eukaryotic translation initiation factor 4E-binding protein 1 (UniProt Accession Q13541) which is a repressor of translational initiation that controls EIF4E activity [66].

## Conclusion

This paper presents a novel predictor Bigram-PGK, which utilizes the feature PSSM + bigram to predict phosphoglycerylation. The underlying evolutionary information in PSSM of protein sequences and its transformation to bigram occurrences appears to be a crucial property in detecting the lysine modification. The use of studied feature in this work and the SVM classifier with polynomial kernel to obtain a decent hyperplane separation was effective to distinguish between the modified and unmodified lysine sites.

## Materials and methods

### Protein dataset

The benchmark dataset used in this work was obtained from the Compendium of Protein Lysine Modifications (CPLM) repository, accessed 1 March 2017 (available at http://cplm.biocuckoo.org) which has now been upgraded to Protein Lysine Modification Database (PLMD). PLMD contains a number of different protein lysine modifications that have been experimentally identified. Phosphoglycerylation dataset obtained was initially prepared by removing sequences which had 40% or higher sequential similarities, which is a widely used level in the literature [40, 67, 68], using the Cd-hit tool [69]. As a result, a total of 91 sequences were attained and in each sequence, there were more than one lysine residue. From these sequences, 3360 lysine residues were found. Three thousand two hundred forty-nine lysines were non-phosphoglycerylated and 111 were phosphoglycerylated.

### Position specific scoring matrix

Evolutionary feature captures how proteins have evolved in relative to its structural, functional and sequential similarities with other protein sequences [70]. PSSM calculates the substitution probability of amino acids in the sequence to all the amino acids of the genetic code. PSSM profiles is a highly revered feature in the area of proteomics [71–73]. The profiles are obtained using PSI-BLAST toolbox [74] which aligns protein sequences to similar sequences stored in protein data bank [75]. The outputs of PSI-BLAST are two matrices with a dimension of L × 20; L being the length of the queried protein sequence and 20 being the 20 amino acids of the genetic code. Of the two matrices, one being log odds and the other the amino acid linear probabilities, the latter was used in this work. The PSSM for the purpose of this work was produced on non-redundant proteins using a threshold value of 0.001 of the PSI-BLAST toolbox with three iterations.

### Feature extraction

**Fig. 1:**
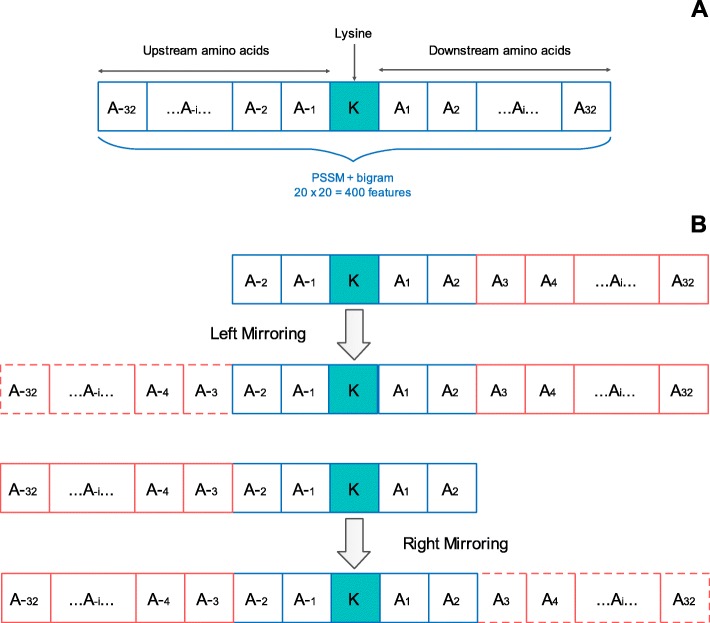
Depiction of lysine residue with its upstream and downstream amino acids. **a** Lysine residue with sufficient neighbors. **b** Scenario of lysine residue with insufficient neighbors on either the upstream or downstream portion

This section deals with the segment sizing for each lysine residue and its corresponding feature extraction. To represent each sample, we have used the evolutionary information of 32 upstream and 32 downstream amino acids to the lysine K portrayed in Fig. 1a. In the cases where lysine residue did not have enough amino acids, either upstream or downstream, the mirror technique [35] was used to create the missing amino acids as shown in Fig. 1b. The segment consisting of 32 upstream and 32 downstream neighboring amino acids of lysine K can be denoted by P as:

6 $$ P=\left\{{A}_{-32},\dots, {A}_{-2},{A}_{-1},K,{A}_1,{A}_2,\dots, {A}_{32}\right\} $$

From eq. (6), the downstream amino acids are represented by An where 1 ≤ *n* ≤ 32 while the upstream by A-n where 1 ≤ *n* ≤ 32. Moreover, it can be seen that a segment consists of a total of 65 amino acids, including the lysine K at the center. The segment P is attached with an experimentally confirmed label of either 1 or a 0 indicating a phosphoglycerylated site or a non-phosphoglycerylated site, respectively. The acquired sub-matrix by segment P describing each lysine was converted to a frequency vector of bigrams (PSSM + bigram) resulting in the matrix of size 20 × 20. Each lysine was then represented by transforming this matrix to a 400 dimensional row vector capturing evolutionary information of the segment P.

### Profile bigrams

The profile bigrams technique has displayed promising results in dealing with discriminatory information [76–79]. For the purpose of explanation, let’s assume that the PSSM of a protein sequence is denoted by a matrix M. Every element in matrix M, indicated by m*ij*, can be said to be the transitional probability of *j*-th amino acid at *i*-th location within the given protein sequence. The segment P, consisting of 65 amino acids (a fraction of the protein sequence), is hence represented by a 65 × 20 feature matrix in which 20 denotes the amino acids of the genetic code. Therefore the PSSM was calculated based on the substitution probabilities of each amino acid in the segment to the 20 amino acids. For the matrix M, its profile bigram is calculated by.

7 $$ {B}_{p,q}={\sum}_{k=1}^{64}{m}_{k,p}{m}_{k+1,q}\;\mathrm{where}\;1\le p\le 20\;\mathrm{and}\;1\le q\le 20 $$

From the above equation, the resulting dimension of matrix B representing the PSSM + bigram is 20 × 20. Finally, matrix B is converted to a 400 dimensional row vector indicated by Eq. (8) which represents the 400 transitional probabilities pertaining to evolutionary information of each lysine residue.

8 $$ F=\left[{B}_{1,1},{B}_{1,2},\dots, {B}_{1,20},{B}_{2,1},{B}_{2,2},\dots, {B}_{2,20},{B}_{20,1},{B}_{20,2},\dots, {B}_{20,20}\right] $$

### Support vector machine

Support vector machine is one of the supervised learning model listed under the topic of machine learning. The algorithm is commonly used in classification and regression applications. It is a discriminative classifier that works by defining a separating hyperplane. Usage of SVM is not only popular in protein problems [20, 80–83], but also in other areas of biology, such as genomes [84, 85]. With a given set of training data, the algorithm produces an optimal hyperplane separating the two classes and for every new data points presented, it is able to categorize based on this defined hyperplane. The data points represent a point in n-dimensional space where n corresponds to the number of features it possesses. These data points of two class problem are not always linearly separable, hence non-linear kernels are used to carry out classification. The non-linear kernels project the nonlinear input space to a higher dimensional space where the classes are linearly separable. For the purpose of this work, LibSVM package on Matlab software was used to carry out the identification of phosphoglycerylated and non-phosphoglycerylated sites using C-SVC type SVM and a polynomial kernel.

## Supplementary information

**Additional file 1. ** MCC values for different segment sizes.

**Additional file 2.** Number of Phosphoglycerylation sites detected by each predictor.

## Data Availability

The datasets used and analyzed during the current study are publically available online at https://github.com/abelavit/Bigram-PGK or www.alok-ai-lab.com.

## Abbreviations

AUC: Area under the ROC curve
MCC: Mathews correlation coefficient
PSSM: Position-specific scoring matrix
PTM: Post-translational modification
SVM: Support vector machine

## References

[1] Huang J, Wang F, Ye M, Zou H (2014). Enrichment and separation techniques for large-scale proteomics analysis of the protein post-translational modifications. J Chromatogr A.

[2] Lanouette S, Mongeon V, Figeys D, Couture JF (2014). The functional diversity of protein lysine methylation. Mol Syst Biol.

[3] Liu Zexian, Wang Yongbo, Gao Tianshun, Pan Zhicheng, Cheng Han, Yang Qing, Cheng Zhongyi, Guo Anyuan, Ren Jian, Xue Yu (2013). CPLM: a database of protein lysine modifications. Nucleic Acids Research.

[4] Chou K-C. An unprecedented revolution in medicinal chemistry driven by the progress of biological science. Curr Top Med Chem. 2017;17(21):2337–58. 10.2174/1568026617666170414145508 28413951

[5] Choudhary C., Kumar C., Gnad F., Nielsen M. L., Rehman M., Walther T. C., Olsen J. V., Mann M. (2009). Lysine Acetylation Targets Protein Complexes and Co-Regulates Major Cellular Functions. Science.

[6] Johansen Morten Bo, Kiemer Lars, Brunak Søren (2006). Analysis and prediction of mammalian protein glycation. Glycobiology.

[7] Lan Fei, Shi Yang (2009). Epigenetic regulation: methylation of histone and non-histone proteins. Science in China Series C: Life Sciences.

[8] Park Jeongsoon, Chen Yue, Tishkoff Daniel X., Peng Chao, Tan Minjia, Dai Lunzhai, Xie Zhongyu, Zhang Yi, Zwaans Bernadette M.M., Skinner Mary E., Lombard David B., Zhao Yingming (2013). SIRT5-Mediated Lysine Desuccinylation Impacts Diverse Metabolic Pathways. Molecular Cell.

[9] Iyer LM, Burroughs AM, Aravind L (2008). Unraveling the biochemistry and provenance of pupylation: a prokaryotic analog of ubiquitination. Biol Direct.

[10] Tan Minjia, Luo Hao, Lee Sangkyu, Jin Fulai, Yang Jeong Soo, Montellier Emilie, Buchou Thierry, Cheng Zhongyi, Rousseaux Sophie, Rajagopal Nisha, Lu Zhike, Ye Zhen, Zhu Qin, Wysocka Joanna, Ye Yang, Khochbin Saadi, Ren Bing, Zhao Yingming (2011). Identification of 67 Histone Marks and Histone Lysine Crotonylation as a New Type of Histone Modification. Cell.

[11] Cheng Z, Tang Y, Chen Y, Kim S, Liu H, Li SS (2009). Molecular characterization of propionyllysines in non-histone proteins. Mol Cell Proteomics.

[12] Szondy Zsuzsa, Korponay-Szabó Ilma, Király Robert, Sarang Zsolt, Tsay Gregory J. (2017). Transglutaminase 2 in human diseases. BioMedicine.

[13] Li S, Iakoucheva LM, Mooney SD, Radivojac P. Loss of post-translational modification sites in disease. Biocomputing 2010: World Scientific; 2010. p. 337–47. 10.1142/9789814295291_0036 PMC2813771 19908386

[14] Liddy KA, White MY, Cordwell SJ (2013). Functional decorations: post-translational modifications and heart disease delineated by targeted proteomics. Genome Med.

[15] Spinelli Francesca Romana, Pecani Arbi, Conti Fabrizio, Mancini Riccardo, Alessandri Cristiano, Valesini Guido (2016). Post-translational modifications in rheumatoid arthritis and atherosclerosis: Focus on citrullination and carbamylation. Journal of International Medical Research.

[16] Ju Zhe, Cao Jun-Zhe, Gu Hong (2016). Predicting lysine phosphoglycerylation with fuzzy SVM by incorporating k-spaced amino acid pairs into Chou׳s general PseAAC. Journal of Theoretical Biology.

[17] Moellering R. E., Cravatt B. F. (2013). Functional Lysine Modification by an Intrinsically Reactive Primary Glycolytic Metabolite. Science.

[18] Bulcun E., Ekici M., Ekici A. (2011). Disorders of glucose metabolism and insulin resistance in patients with obstructive sleep apnoea syndrome. International Journal of Clinical Practice.

[19] Kolwicz SC, Tian R (2011). Glucose metabolism and cardiac hypertrophy. Cardiovasc Res.

[20] López Y, Sharma A, Dehzangi A, Lal SP, Taherzadeh G, Sattar A (2018). Success: evolutionary and structural properties of amino acids prove effective for succinylation site prediction. BMC Genomics.

[21] Ju Zhe, He Jian-Jun (2017). Prediction of lysine propionylation sites using biased SVM and incorporating four different sequence features into Chou’s PseAAC. Journal of Molecular Graphics and Modelling.

[22] Xu Y, Ding Y-X, Ding J, Wu L-Y, Xue Y (2016). Mal-Lys: prediction of lysine malonylation sites in proteins integrated sequence-based features with mRMR feature selection. Sci Rep.

[23] Xiang Q, Feng K, Liao B, Liu Y, Huang G. Prediction of lysine Malonylation sites based on Pseudo amino acid. Comb Chem High Throughput Screen. 2017;20(7):622–8. 10.2174/1386207320666170314102647 28292251

[24] Du Yipeng, Zhai Zichao, Li Ying, Lu Ming, Cai Tanxi, Zhou Bo, Huang Lei, Wei Taotao, Li Tingting (2016). Prediction of Protein Lysine Acylation by Integrating Primary Sequence Information with Multiple Functional Features. Journal of Proteome Research.

[25] Qiu Wang-Ren, Xiao Xuan, Lin Wei-Zhong, Chou Kuo-Chen (2014). iUbiq-Lys: prediction of lysine ubiquitination sites in proteins by extracting sequence evolution information via a gray system model. Journal of Biomolecular Structure and Dynamics.

[26] Hou T, Zheng G, Zhang P, Jia J, Li J, Xie L (2014). LAceP: lysine acetylation site prediction using logistic regression classifiers. PLoS One.

[27] Jia Jianhua, Zhang Liuxia, Liu Zi, Xiao Xuan, Chou Kuo-Chen (2016). pSumo-CD: predicting sumoylation sites in proteins with covariance discriminant algorithm by incorporating sequence-coupled effects into general PseAAC. Bioinformatics.

[28] Qiu W-R, Sun B-Q, Xiao X, Xu Z-C, Jia J-H, Chou K-C (2017). iKcr-PseEns: identify lysine crotonylation sites in histone proteins with pseudo components and ensemble classifier. Genomics.

[29] Ju Z, Gu H (2016). Predicting pupylation sites in prokaryotic proteins using semi-supervised self-training support vector machine algorithm. Anal Biochem.

[30] Bakhtiarizadeh MR, Moradi-Shahrbabak M, Ebrahimi M, Ebrahimie E (2014). Neural network and SVM classifiers accurately predict lipid binding proteins, irrespective of sequence homology. J Theor Biol.

[31] Liu Y, Wang M, Xi J, Luo F, Li A (2018). PTM-ssMP: a web server for predicting different types of post-translational modification sites using novel site-specific modification profile. Int J Biol Sci.

[32] Wang B, Wang M, Li A (2017). Prediction of post-translational modification sites using multiple kernel support vector machine. PeerJ.

[33] Fan W, Xu X, Shen Y, Feng H, Li A, Wang M (2014). Prediction of protein kinase-specific phosphorylation sites in hierarchical structure using functional information and random forest. Amino Acids.

[34] Chandra A, Sharma A, Dehzangi A, Ranganathan S, Jokhan A, Chou K-C (2018). PhoglyStruct: prediction of phosphoglycerylated lysine residues using structural properties of amino acids. Sci Rep.

[35] Dehzangi A, López Y, Lal SP, Taherzadeh G, Michaelson J, Sattar A (2017). PSSM-Suc: accurately predicting succinylation using position specific scoring matrix into bigram for feature extraction. J Theor Biol.

[36] Chou K-C, Shen H-B (2007). Recent progress in protein subcellular location prediction. Anal Biochem.

[37] Jia J, Liu Z, Xiao X, Liu B, Chou K-C (2016). iSuc-PseOpt: identifying lysine succinylation sites in proteins by incorporating sequence-coupling effects into pseudo components and optimizing imbalanced training dataset. Anal Biochem.

[38] Xu Y, Ding Y-X, Ding J, Wu L-Y, Deng N-Y (2015). Phogly–PseAAC: prediction of lysine phosphoglycerylation in proteins incorporating with position-specific propensity. J Theor Biol.

[39] Liu L-M, Xu Y, Chou K-C (2017). iPGK-PseAAC: identify lysine phosphoglycerylation sites in proteins by incorporating four different tiers of amino acid pairwise coupling information into the general PseAAC. Med Chem.

[40] Dehzangi A, López Y, Lal SP, Taherzadeh G, Sattar A, Tsunoda T (2018). Improving succinylation prediction accuracy by incorporating the secondary structure via helix, strand and coil, and evolutionary information from profile bigrams. PLoS One.

[41] López Y, Dehzangi A, Lal SP, Taherzadeh G, Michaelson J, Sattar A (2017). SucStruct: prediction of succinylated lysine residues by using structural properties of amino acids. Anal Biochem.

[42] Liu Z, Xiao X, Qiu W-R, Chou K-C (2015). iDNA-methyl: identifying DNA methylation sites via pseudo trinucleotide composition. Anal Biochem.

[43] Jia J, Liu Z, Xiao X, Liu B, Chou K-C (2016). iPPBS-opt: a sequence-based ensemble classifier for identifying protein-protein binding sites by optimizing imbalanced training datasets. Molecules.

[44] Chen W, Feng P, Ding H, Lin H, Chou K-C (2015). iRNA-methyl: identifying N6-methyladenosine sites using pseudo nucleotide composition. Anal Biochem.

[45] Liu B, Fang L, Wang S, Wang X, Li H, Chou K-C (2015). Identification of microRNA precursor with the degenerate K-tuple or Kmer strategy. J Theor Biol.

[46] Ding Hui, Deng En-Ze, Yuan Lu-Feng, Liu Li, Lin Hao, Chen Wei, Chou Kuo-Chen (2014). iCTX-Type: A Sequence-Based Predictor for Identifying the Types of Conotoxins in Targeting Ion Channels. BioMed Research International.

[47] Xiao X, Min J-L, Lin W-Z, Liu Z, Cheng X, Chou K-C (2015). iDrug-target: predicting the interactions between drug compounds and target proteins in cellular networking via benchmark dataset optimization approach. J Biomol Struct Dyn.

[48] Chou K-C, Zhang C-T (1995). Prediction of protein structural classes. Crit Rev Biochem Mol Biol.

[49] Chou KC (2001). Prediction of protein cellular attributes using pseudo-amino acid composition. Proteins.

[50] Hajisharifi Z, Piryaiee M, Beigi MM, Behbahani M, Mohabatkar H (2014). Predicting anticancer peptides with Chou’ s pseudo amino acid composition and investigating their mutagenicity via Ames test. J Theor Biol.

[51] Huang X, Wang Y, Yan W, Smith C, Ye Z, Wang J (2015). Production of gene-corrected adult Beta globin protein in human erythrocytes differentiated from patient i PSC s after genome editing of the sickle point mutation. Stem Cells.

[52] De Groot C, Zonneveld D, De Laaf R, Dingemanse M, Mooren P, Moorman A (1986). Developmental and hormonal regulation of carbamoyl-phosphate synthase gene expression in rat liver: evidence for control mechanisms at different levels in the perinatal period. Biochim Biophys Acta.

[53] Inoue H, Randazzo PA (2007). Arf GAPs and their interacting proteins. Traffic.

[54] Aitken A. 14-3-3 proteins: a historic overview. In Seminars in cancer biology. Academic Press; 2006;16(3):162–72. 10.1016/j.semcancer.2006.03.005 16678438

[55] Melnikov S, Ben-Shem A, De Loubresse NG, Jenner L, Yusupova G, Yusupov M (2012). One core, two shells: bacterial and eukaryotic ribosomes. Nat Struct Mol Biol.

[56] Ladomery M, Dellaire G (2002). Multifunctional zinc finger proteins in development and disease. Ann Hum Genet.

[57] Volgraf M, Gorostiza P, Numano R, Kramer RH, Isacoff EY, Trauner D (2005). Allosteric control of an ionotropic glutamate receptor with an optical switch. Nat Chem Biol.

[58] Naslavsky N, Caplan S (2005). C-terminal EH-domain-containing proteins: consensus for a role in endocytic trafficking, EH?. J Cell Sci.

[59] Ikegawa S, Isomura M, Koshizuka Y, Nakamura Y (1999). Cloning and characterization of human and mouse PROSC (proline synthetase co-transcribed) genes. J Hum Genet.

[60] Reshetnikov AS, Rozova ON, Khmelenina VN, Mustakhimov II, Beschastny AP, Murrell JC (2008). Characterization of the pyrophosphate-dependent 6-phosphofructokinase from Methylococcus capsulatus Bath. FEMS Microbiol Lett.

[61] Kavanagh KL, Guo K, Dunford JE, Wu X, Knapp S, Ebetino FH (2006). The molecular mechanism of nitrogen-containing bisphosphonates as antiosteoporosis drugs. Proc Natl Acad Sci.

[62] Palmieri L, Pardo B, Lasorsa F, Del Arco A, Kobayashi K, Iijima M (2001). Citrin and aralar1 are Ca2+−stimulated aspartate/glutamate transporters in mitochondria. EMBO J.

[63] Richard JP (1991). Kinetic parameters for the elimination reaction catalyzed by triosephosphate isomerase and an estimation of the reaction's physiological significance. Biochemistry.

[64] Toyoshima I, Yu H, Steuer ER, Sheetz MP (1992). Kinectin, a major kinesin-binding protein on ER. J Cell Biol.

[65] Tunio SA, Oldfield NJ, Berry A, Ala’Aldeen DA, Wooldridge KG, Turner DP (2010). The moonlighting protein fructose-1, 6-bisphosphate aldolase of Neisseria meningitidis: surface localization and role in host cell adhesion. Mol Microbiol.

[66] Pause A, Belsham GJ, Gingras A-C, Donzé O, Lin T-A, Lawrence JC (1994). Insulin-dependent stimulation of protein synthesis by phosphorylation of a regulator of 5′-cap function. Nature.

[67] Shi S-P, Qiu J-D, Sun X-Y, Suo S-B, Huang S-Y, Liang R-P (2012). PMeS: prediction of methylation sites based on enhanced feature encoding scheme. PLoS One.

[68] Ju Z, He J-J (2018). Prediction of lysine glutarylation sites by maximum relevance minimum redundancy feature selection. Anal Biochem.

[69] Li W, Godzik A (2006). Cd-hit: a fast program for clustering and comparing large sets of protein or nucleotide sequences. Bioinformatics.

[70] Dehzangi Abdollah, Paliwal Kuldip, Lyons James, Sharma Alok, Sattar Abdul (2013). Exploring Potential Discriminatory Information Embedded in PSSM to Enhance Protein Structural Class Prediction Accuracy. Pattern Recognition in Bioinformatics.

[71] Le NQK, Ho QT, Ou YY (2017). Incorporating deep learning with convolutional neural networks and position specific scoring matrices for identifying electron transport proteins. J Comput Chem.

[72] Le NQK, Huynh T-T, Yapp EKY, Yeh H-Y (2019). Identification of clathrin proteins by incorporating hyperparameter optimization in deep learning and PSSM profiles. Comput Methods Prog Biomed.

[73] Ho Q-T, Ou Y-Y (2018). Classifying the molecular functions of Rab GTPases in membrane trafficking using deep convolutional neural networks. Anal Biochem.

[74] Altschul SF, Madden TL, Schäffer AA, Zhang J, Zhang Z, Miller W (1997). Gapped BLAST and PSI-BLAST: a new generation of protein database search programs. Nucleic Acids Res.

[75] Berman H, Westbrook J, Feng Z, Gilliland G, Bhat T, Weissig H (2000). The protein data Bank nucleic acids research.

[76] Sharma A, Lyons J, Dehzangi A, Paliwal KK (2013). A feature extraction technique using bi-gram probabilities of position specific scoring matrix for protein fold recognition. J Theor Biol.

[77] Dehzangi A, Heffernan R, Sharma A, Lyons J, Paliwal K, Sattar A (2015). Gram-positive and gram-negative protein subcellular localization by incorporating evolutionary-based descriptors into Chou’ s general PseAAC. J Theor Biol.

[78] Paliwal KK, Sharma A, Lyons J, Dehzangi A (2014). A tri-gram based feature extraction technique using linear probabilities of position specific scoring matrix for protein fold recognition. IEEE Trans Nanobioscience.

[79] Sharma R, Dehzangi A, Lyons J, Paliwal K, Tsunoda T, Sharma A (2015). Predict gram-positive and gram-negative subcellular localization via incorporating evolutionary information and physicochemical features into Chou's general PseAAC. IEEE Trans Nanobioscience.

[80] Sharma R, Raicar G, Tsunoda T, Patil A, Sharma A (2018). OPAL: Prediction of MoRF regions in intrinsically disordered protein sequences. Bioinformatics.

[81] Chandra AA, Sharma A, Dehzangi A, Tsunoda T (2019). EvolStruct-Phogly: incorporating structural properties and evolutionary information from profile bigrams for the phosphoglycerylation prediction. BMC Genomics.

[82] Sharma R, Sharma A, Raicar G, Tsunoda T, Patil A (2019). OPAL+: length-specific MoRF prediction in intrinsically disordered protein sequences. Proteomics.

[83] Sharma R, Bayarjargal M, Tsunoda T, Patil A, Sharma A (2018). MoRFPred-plus: computational identification of MoRFs in protein sequences using physicochemical properties and HMM profiles. J Theor Biol.

[84] Le NQK, Yapp EKY, Ho Q-T, Nagasundaram N, Ou Y-Y, Yeh H-Y (2019). iEnhancer-5Step: identifying enhancers using hidden information of DNA sequences via Chou's 5-step rule and word embedding. Anal Biochem.

[85] Le Nguyen Quoc Khanh (2019). iN6-methylat (5-step): identifying DNA N6-methyladenine sites in rice genome using continuous bag of nucleobases via Chou’s 5-step rule. Molecular Genetics and Genomics.

