# Supplementary material for: Bigram-PGK: phosphoglycerylation prediction using the technique of bigram probabilities of position specific scoring matrix
Source: BMC Mol Cell Biol. 2019 Dec 20;20(Suppl 2):57. doi: 10.1186/s12860-019-0240-1 (PMC6923822; doi:10.1186/s12860-019-0240-1)
Supplement: Supplementary file 1 — Additional file 1. MCC values for different segment sizes. [file 12860_2019_240_MOESM1_ESM.pdf]

Bigram-PGK: phosphoglycerylation prediction using the technique of bigram probabilities of position specific scoring matrix

Abel Chandra[5,*], Alok Sharma[1,2,3,5,7,*], Abdollah Dehzangi[4], Daichi Shigemizu[2,3,6,7], and Tatsuhiko Tsunoda[2,3,7]

[1] Institute for Integrated and Intelligent Systems, Griffith University, Brisbane, QLD-4111, Australia
[2] Department of Medical Science Mathematics, Medical Research Institute, Tokyo Medical and Dental University (TMDU), Tokyo, 113-8510, Japan
[3] Laboratory for Medical Science Mathematics, RIKEN Center for Integrative Medical Sciences, Yokohama, 230-0045, Kanagawa, Japan
[4] Department of Computer Science, Morgan State University, Baltimore, Maryland, USA
[5] School of Engineering and Physics, Faculty of Science Technology and Environment, University of the South Pacific, Suva, Fiji
[6] Medical Genome Center, National Center for Geriatrics and Gerontology, Obu, Aichi 474-8511, Japan
[7] CREST, JST, Tokyo, 102-8666, Japan
[*] Corresponding authors
abelavit@gmail.com, alok.sharma@griffith.edu.au

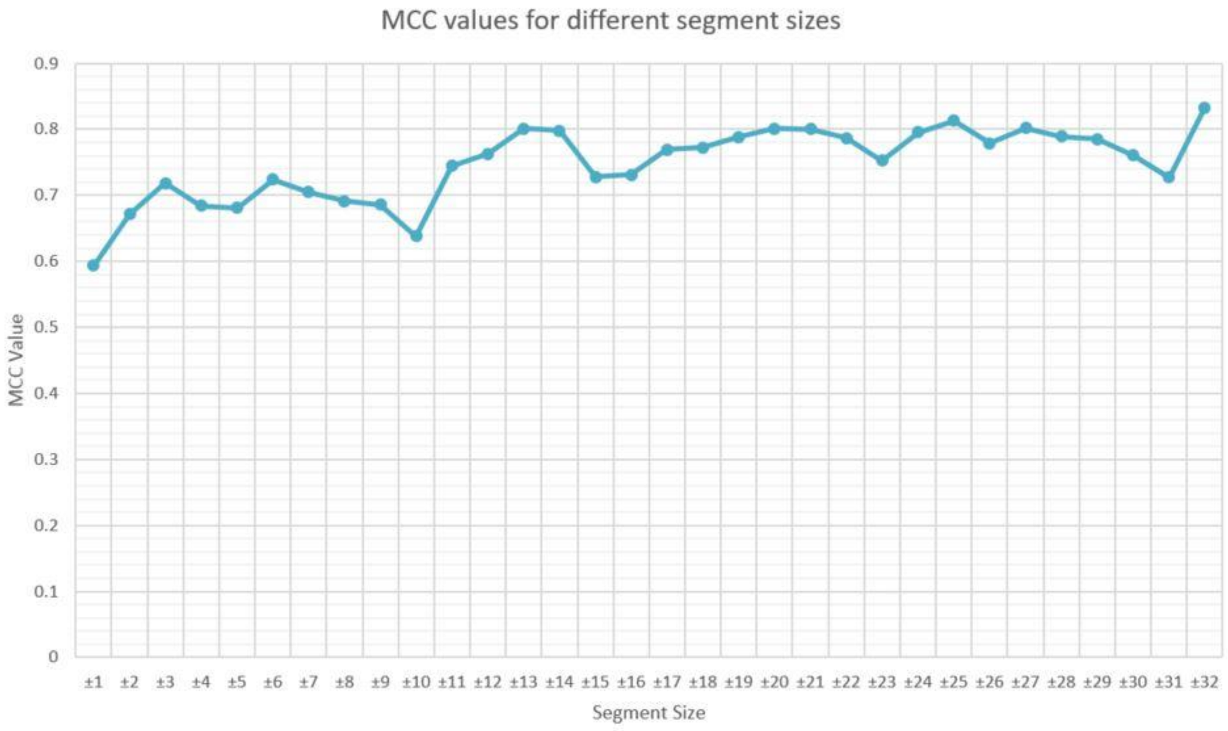

Segment	MCC
±1	0.5933
±2	0.6714
±3	0.7184
±4	0.6843
±5	0.6808
±6	0.7238
±7	0.705
±8	0.6912
±9	0.686
±10	0.6377
±11	0.7449
±12	0.763
±13	0.8012
±14	0.798
±15	0.7279
±16	0.7308

Segment	MCC
±17	0.7694
±18	0.7724
±19	0.7885
±20	0.8015
±21	0.8001
±22	0.7871
±23	0.753
±24	0.7957
±25	0.813
±26	0.7785
±27	0.8018
±28	0.7889
±29	0.785
±30	0.7608
±31	0.7272
±32	**0.833**
