# Supplementary material for: Bigram-PGK: phosphoglycerylation prediction using the technique of bigram probabilities of position specific scoring matrix
Source: BMC Mol Cell Biol. 2019 Dec 20;20(Suppl 2):57. doi: 10.1186/s12860-019-0240-1 (PMC6923822; doi:10.1186/s12860-019-0240-1)
Supplement: Supplementary file 2 — Additional file 2. Number of Phosphoglycerylation sites detected by each predictor. [file 12860_2019_240_MOESM2_ESM.pdf]

Bigram-PGK: phosphoglycerylation prediction using the technique of bigram probabilities of position specific scoring matrix

Abel Chandra[5,*], Alok Sharma[1,2,3,5,7,*], Abdollah Dehzangi[4], Daichi Shigemizu[2,3,6,7], and Tatsuhiko Tsunoda[2,3,7]

[1] Institute for Integrated and Intelligent Systems, Griffith University, Brisbane, QLD-4111, Australia
[2] Department of Medical Science Mathematics, Medical Research Institute, Tokyo Medical and Dental University (TMDU), Tokyo, 113-8510, Japan
[3] Laboratory for Medical Science Mathematics, RIKEN Center for Integrative Medical Sciences, Yokohama, 230-0045, Kanagawa, Japan
[4] Department of Computer Science, Morgan State University, Baltimore, Maryland, USA
[5] School of Engineering and Physics, Faculty of Science Technology and Environment, University of the South Pacific, Suva, Fiji
[6] Medical Genome Center, National Center for Geriatrics and Gerontology, Obu, Aichi 474-8511, Japan
[7] CREST, JST, Tokyo, 102-8666, Japan
[*] Corresponding authors
abelavit@gmail.com, alok.sharma@griffith.edu.au

Table 1. Number of Phosphoglycerylation sites detected by each predictor.

Protein ID	Sites	Bigram-PGK	Phogly_PseAAC	CKSAAP_PhoglySite	iPGK_PseAAC
'Q3UJB0'	1	0	1	1	0
E9QMI7'	1	1	0	0	0
P47857'	1	1	1	1	1
Q920E5'	1	1	1	1	1
'Q9C026'	1	1	1	1	0
'P26040'	1	0	1	0	0
Q3UL97'	1	1	1	1	1
'P11983'	1	1	1	1	0
Q8C196'	4	4	2	3	1
'Q9BYV9'	1	1	0	1	1
'P51814'	1	0	0	1	1
'Q9H1E3'	1	0	1	1	0
'O88844'	1	1	0	1	1
'J3KMM5'	1	0	0	1	0
P01942'	3	3	2	3	3
'P11881'	1	1	0	1	0
A2AQC3'	1	1	1	1	1
Q9QXX4'	1	1	1	1	1
'P23881'	1	1	0	1	0
P60174'	1	1	1	1	1
F8WIT2'	3	3	2	3	2
Q86UP2'	1	1	1	1	1
P18894'	2	2	2	2	1

A6ZI44'	1	1	1	1	1
'P49722'	1	1	0	1	0
'Q5RL73'	1	1	0	1	0
'P07724'	1	1	1	0	0
Q9EQF5'	1	1	1	1	1
'P22752'	1	0	0	1	0
'D3YU05'	5	5	4	4	0
'J3QNG0'	1	1	1	1	0
'D3Z6C3'	1	0	0	0	0
'Q9D020'	1	1	1	1	1
'P62908'	1	1	0	1	1
Q9Y619'	1	1	1	1	1
A2A5N1'	1	1	0	0	0
'Q3UNI1'	1	1	1	0	0
'Q9DAY2'	1	1	1	0	1
E9PZS8'	1	1	1	1	1
'D3Z563'	1	1	1	1	0
'P11214'	1	1	0	1	1
E9QNN1'	1	1	1	1	1
J3KRX8'	1	1	1	1	1
Q5JSZ5'	2	2	1	2	1
'E7EP94'	1	1	0	0	0
'Q8BH04'	1	0	0	1	0
A8DUK4'	4	3	4	3	1
'P99028'	1	1	1	1	0
'P16015'	1	1	1	1	0
'I7HPV9'	1	1	0	1	0
D3Z041'	2	2	1	2	1
E9Q1V0'	1	1	1	1	1
'C9J5S8'	1	1	1	1	0
'P06733'	2	2	2	2	1
'B4DPF6'	1	1	1	1	0
'B1AU42'	1	1	1	0	1
O70250'	1	1	1	1	1
'Q64FW2'	1	1	1	1	0
O09172'	1	1	1	1	1
'P68104'	1	1	0	1	0
'Q93092'	1	1	1	0	0
Q8BMS1'	1	1	1	1	1
'P12790'	1	0	1	1	0
H3BTN5'	1	1	1	1	1
P62900'	1	1	0	0	0
Q13541'	1	1	1	1	1

P26443'	1	1	1	1	1
'P12710'	1	0	0	1	1
B1AS29'	1	0	1	1	1
P47806'	1	1	0	0	0
'F6UB20'	1	1	1	1	0
Q9Y597'	1	1	1	1	1
Q9EQP2'	1	0	1	1	1
'E9Q070'	1	1	1	0	0
'P48962'	2	2	1	2	1
'Q9EP89'	1	1	1	1	1
'P16879'	1	1	0	1	1
P14174'	1	1	1	1	1
'Q8VD72-2'	1	1	1	0	0
E9Q3T0'	1	1	0	0	0
'J3QMG3'	2	2	2	2	0
'G3UYJ7'	1	0	1	1	0
'F6YLP3'	1	1	1	1	1
'Q9DB16'	1	0	1	1	0
'Q91V76'	1	1	1	1	1
'P49006'	1	1	0	1	1
'P35585'	1	1	1	1	0
'P62814'	1	1	1	1	1
Q80ZV3'	1	0	0	0	0
'B8ZZL8'	1	0	1	1	0
'Q6PKG0'	1	1	0	1	0
